# Collagen type III alpha I is a gastro-oesophageal reflux disease susceptibility gene and a male risk factor for hiatus hernia

**DOI:** 10.1136/gut.2008.167353

**Published:** 2009-04-26

**Authors:** B Åsling, J Jirholt, P Hammond, M Knutsson, A Walentinsson, G Davidson, L Agreus, A Lehmann, M Lagerström-Fermer

**Affiliations:** 1 AstraZeneca R&D, Mölndal, Sweden; 2 Women’s & Children’s Hospital, Gastroenterology Unit, North Adelaide, Australia; 3 Center for Family and Community Medicine, Karolinska Institutet, Huddinge, Sweden

## Abstract

**Background and objectives:**

Gastro-oesophageal reflux disease (GORD) is a common gastrointestinal disorder with a genetic component. Our aim was to identify genetic factors associated with GORD.

**Patients and methods:**

Four separate patient cohorts were analysed using a step-wise approach. (1) Whole genome linkage analysis was performed in 36 families. (2) Candidate genes were tested for GORD association in a trio cohort. (3) Genetic association was replicated in a case–control cohort. We also investigated genetic association to hiatus hernia (HH). (4) Protein expression was analysed in oesophageal biopsies.

**Results:**

A region on chromosome 2, containing collagen type III alpha 1 (COL3A1), was identified (LOD = 3.3) in families with dominant transmission of GORD, stratified for hiatus hernia (HH). COL3A1 showed significant association with GORD in an independent paediatric trio cohort (p_corr_ = 0.003). The association was male specific (p_corr_ = 0.018). The COL3A1 association was replicated in an independent adult case control cohort (p_corr_ = 0.022). Moreover, male specific association to HH (p_corr_ = 0.019) was found for a SNP not associated to GORD. Collagen type III protein was more abundant in oesophageal biopsies from male patients with GORD (p = 0.03).

**Conclusion:**

COL3A1 is a disease-associated gene in both paediatric and adult GORD. Furthermore, we show that COL3A1 is genetically associated with HH in adult males. The GORD- and HH-associated alleles are different, indicating two separate mechanisms leading to disease. Our data provides new insight into GORD aetiology, identifying a connective tissue component and indicating a tissue remodelling mechanism in GORD. Our results implicate gender differences in the genetic risk for both for GORD and HH.

Gastro-oesophageal reflux disease (GORD) is characterised by a retrograde movement of stomach contents into the oesophagus, leading to symptoms such as heartburn and regurgitation.^1^ In severe cases, the disease causes tissue erosion and inflammation in the oesophageal mucosal lining. GORD is increasingly common in the Western world, with a prevalence of 25–40% in population-based studies.^2^ ^3^ Patients suffering from GORD have a severely impaired quality of life,^4^ and the cost to the society is substantial.^5^

The establishment of diagnostic criteria for GORD is made complicated by the fact that patients with GORD represent a heterogeneous patient group. Apart from heartburn and acid regurgitation, diverse additional symptoms, including extra-oesophageal manifestations, have been recognised as important disease components.^2^ Today, diagnosis is generally based on symptomatic presentation complemented by endoscopic and pH probe findings together with data from validated multidimensional questionnaires.^6^ Less than half of the patients suffer from erosive reflux disease, characterised by mucosal damage in the oesophagus. However, the majority of patients have non-erosive reflux disease, and experience typical GORD symptoms without visible oesophageal injury.^7^

Epidemiological studies have pinpointed a number of lifestyle-related factors affecting the disease.^2^ One such risk factor is hiatus hernia (HH), a condition characterised by a protrusion of the upper part of the stomach into the thorax through a tear or weakness in the diaphragm.^8^ ^9^ HH leads to reflux episodes through an attenuation of the pressure barrier, constituted by the lower oesophageal sphincter in conjunction with the diaphragm.^10^ Interestingly, there are data indicating a genetic contribution to the development of HH.^11^

The age of onset of GORD is variable and many individuals develop the disease during childhood. GORD is the most common oesophageal disorder of children, affecting about 11% of all infants during their first year of life.^12^ It has been suggested that adult GORD may sometimes originate in childhood.^3^ ^13^^–^16 The disease aetiology is further complicated by a substantial genetic contribution as shown by; familial clustering,^17^ autosomal dominant familial transmission of disease,^18^ ^19^ as well as twin studies.^20^^–^22 Hu and colleagues addressed this and identified a linked region on chromosome 13q14 in families with severe paediatric GORD.^18^ Orenstein and colleagues, however, failed to replicate this linkage finding in a different GORD family material.^19^ Although this region is relatively well defined, subsequent work has, so far, not led to the identification of a disease susceptibility gene on chromosome 13q14.^23^

The aim of this study was to identify genes associated with GORD and to investigate if these genes are shared between paediatric and adult forms of the disease. To address this, four separate patient cohorts were examined in a step-wise manner. First, genome-wide linkage analysis was carried out in families displaying an apparently dominant inheritance of the disease. Next, gene association analyses were performed in a paediatric trio cohort, followed by replication of results in an adult case–control cohort. Lastly, protein levels were examined in oesophageal biopsies from adult patients and healthy controls.

## METHODS

### Patient collections

Informed consent was obtained before enrolment. All data and DNA/tissue samples were coded. Ethical approval was obtained for all patient collections.

### Families

Enrolment of patients was done at the Gastroenterology Unit at Women’s and Children’s Hospital (WCH, Adelaide, Australia), from 2001 to 2005 by identifying children (age up to 18) diagnosed with GORD having at least one of the following criteria within the last 5 years: an abnormal finding from endoscopic examination or 24 h oesophageal pH test or having been subject to fundoplication. Probands were identified by examination of patient records at WCH and by contacting the local GORD support association. Parents of the proband were contacted and the family was enrolled if a positive family history of GORD could be shown. Disease status was assessed via physician diagnosis often with results of previously performed investigations.^24^^–^26 Patients with a medical condition known to predispose to GORD were excluded. HH was identified through previously performed endoscopic examination and/or radiological contrast studies.

### Trios

The trio cohort consists of paediatric patients diagnosed with GORD between 3 months and 17 years of age without conditions predisposing to GORD, and their parents. The diagnosis of GORD relied on the following: a paediatric gastroenterologist’s evaluation of symptoms to be consistent with GORD, in addition to either endoscopic, histological or pH probe determined acid exposure consistent with GORD and/or a definite and significant improvement in symptoms of GORD shortly after commencing anti-reflux treatment. Previous anti-reflux surgery was also considered as evidence of GORD. Patients were identified from databases of endoscopy, pH probe and outpatient diagnoses from the Gastroenterology Unit at WCH.

### Adult GORD case–control cohort (Extended Kalixanda)

The Kalixanda cohort is a collection of patients from northern Sweden where epidemiological factors related to GORD have been investigated.^27^ ^28^ We extended this cohort with 100 patients with GORD from local clinics, resulting in a new cohort, referred to as the extended Kalixanda cohort. All individuals went through an endoscopic examination together with a written questionnaire to diagnose GORD and HH.^28^

### Adult GORD case–control cohort (EsoNerd)

EsoNerd is a collection of 30 adult GORD cases (18 males) and eight healthy controls (four males) from western Sweden. Oesophageal biopsies were obtained and used for gene chip expression studies and immunohistochemistry (manuscript in preparation, Pierrou *et al*).

Healthy volunteers and GORD patients went through pH metric and endoscopic examinations together with an evaluation of symptoms by a gastroenterologist. Symptom-free individuals with negative pH metric and endoscopic examination were chosen as control individuals.

Individuals with GORD symptoms with positive acid exposure measurements were defined as GORD patients.

### Techniques

#### DNA extraction

DNA was obtained from blood or buccal swabs using Qiagen Blood DNA extraction kit (Qiagen, Valencia, California, USA) or was whole genome amplified using GenomPhi (Amersham Biosciences, Uppsala, Sweden) according to the manufacturer’s protocol. In cases where the DNA amount was limited, whole genome amplification was performed by Molecular Staging (Denver, Colorado, USA) through their Repli-g service.

#### Genotyping

Microsatellite genotyping was performed in 419 familial DNA samples using ABI Prism Linkage Mapping set v2.5 HD5 for DNA fragment analysis (Applied Biosystems, Foster City, California, USA). Additional microsatellites were amplified using public primer sequences. The forward primer was ordered 5′-fluorophore labelled (Sigma–Genosys, Cambridge, UK) while the reverse primer was optimised.^29^ Size fractioning of DNA fragments was done in ABI 3700 or 3730 DNA analysers (Applied Biosystems). Alleles were called using the Genotyper v 3.0 software (Applied Biosystems). The mean distance between adjacent markers was 4.32 centimorgan (cM) (SD 2.65); range, 0–14.78. SNP genotyping was performed using the TaqMan assay (Applied Biosystems) and detection in ABI 7900HT (Applied Biosystems) according to the manufacturer’s recommendations, apart from reducing the total reaction volume to 2.5 μl. All data was analysed with Sequence Detection Systems software v2.1 (Applied Biosystems).

#### Sequencing

Sequencing was performed using the Applied Biosystems 3700 or 3730 automated DNA sequencers (Applied Biosystems) according to the manufacturer’s recommendation. Sequencing was performed from both directions and analysis was performed using Sequencher 4.6 (Genecodes, Ann Arbor, Michigan, USA) and compared with the public genomic sequence (ENST00000304636, Ensembl release 49). Sequence differences were manually checked and remaining inconsistencies were resequenced.

#### Immunohistochemistry

Paraffin-embedded oesophageal tissue sections were treated according to standard protocols for rehydration and antigen retrieval. Immunohistochemistry was performed using the collagen type III specific antibody CL50311AP (Cederlane Laboratories, Burlington, Ontario, Canada) diluted 1:1500. A biotinylated secondary antibody was used (DakoCytomation, Glostrup, Denmark) and the immunoreaction was detected using the chromogen DAB kit: peroxidase/DAB+, Rabbit/Mouse (DAKOCytomation Code K5001). Digital images from the tissue sections were captured and computerised planimetry was performed using the program Picsara (Euromed Networks, Stockholm, Sweden). The area of interest (total area) was set to the epithelial layer, defined to be between the lumen and the muscularis mucosae. The strong positive immunoreactivation seen in the submucosa of the biopsies was used as an internal positive control for the experiment. The staining in the submucosa was used to confine the positive reaction using the upper and lower threshold values (Picsara). The identified setting was then used in all images.

### Statistics

#### Quality control of familial genotype data

Mendelian inconsistencies were detected using PedCheck (v.1.1).^30^ Markers with a large number of errors were either re-analysed or excluded. Individual erroneous genotypes were either rechecked or discarded. Hardy–Weinberg testing was performed in Pedstats (v.0.6.4.^31^ Finally, Merlin (v.0.10.2)^32^ and, in the case of the largest family, SimWalk2 (v.2.91)^33^^–^35 were used to find genotypes due to unlikely recombinations. Although such genotypes were not automatically discarded, it was ascertained that they did not influence the linkage peaks.

#### Quality control of trio and extended Kalixanda genotype data

Mendelian inconsistencies in trio genotypes were detected using PedCheck (v.1.1). Hardy–Weinberg testing as well as calculation of descriptive statistics were performed in both Pedstats (v.0.6.4) and Haploview v.3.2^36^ for both the Trios and the extended Kalixanda cohort.

#### Linkage analysis

Genetic map positions used in the linkage analysis were obtained from the Decode map.^37^ Markers that did not have an assigned position were extrapolated into the map based on physical distance to the flanking markers. Family-wise multipoint logarithm of the odds ratio (LOD) score curves were calculated for all autosomal chromosomes and the X chromosome using Genehunter v.2.1^38^ ^39^ and, in the case of the largest family, SimWalk2 (v.2.91), assuming a dominant mode of inheritance. Single point LOD scores were calculated and compared with the multipoint LOD scores for data consistency. Linkages are reported according to established guidelines.^40^ Sensitivity analysis was performed by (1) varying the parameters in the dominant model, (2) assessing an additive mode of inheritance, and (3) performing non parametric analysis. These analyses provided no further information and the results are thus omitted.

#### Association analysis trios

Single SNPs TDT analysis was performed in Genehunter (v.2.1) and Haploview (v.3.2). TDT analysis based on haplotypes was performed in Genehunter (v.2.1). p values adjusted for multiple testing (p_corr_) were assessed by permutations between transmitted and untransmitted haplotypes. For single SNPs, Haploview (v.3.2) was used, whereas a SAS (v.8.2) macro was written to obtain adjusted p values for haplotypes, using the transmit/untransmit-files provided by Genehunter.

#### Association analysis extended Kalixanda

Case–control analysis based on single SNPs was performed in SAS (v.8.2) using Fisher’s exact test. p values adjusted for multiple testing were obtained by permutations of case–control status. Haplotype analysis was performed in WHAP (v.2.06).^41^

#### Immunohistochemistry

The difference of COL3A1 immunostaining in healthy volunteers and patients with GORD from the EsoNerd study was tested using the Wilcoxon rank sum test in R (v.2.4.1). (http://www.R-project.org)

## RESULTS

### Linkage analysis

**Figure 1:**
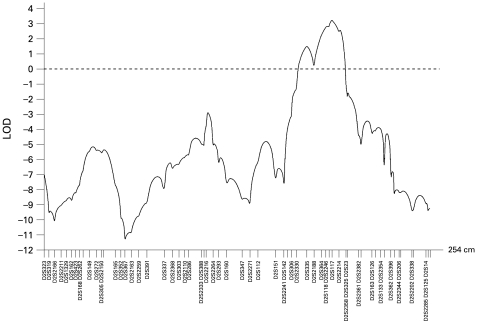
Logarithm of the odds ratio (LOD) curve for chromosome 2. Families were stratified for the presence of hiatus hernia and the absence of Barrett’s oesophagus. One single linkage region was identified with a LOD score of 3.3. The genomic region defined by maximum LOD – 1 is roughly 35 Mbp.

The four patient collections used in this study are summarised in table 1. In the initial analysis we investigated the existence of disease-linked chromosomal regions using the entire family collection. We collected 36 families, encompassing a total of 504 individuals. Of these, 237 were affected with GORD and 99 were assigned with unknown disease status. The families were composed of three generations for 22 families, four generations for 12 families and five generations for two families. Based on the assumption that different genetic factors are segregating in different subsets of the families, the material was stratified so that families where HH was present and Barrett’s oesophagus was absent were selected for further analysis. In this group of families, we were able to identify a common linked region on chromosome 2q24–q33 with a LOD of 3.3 (fig 1). The size of the region was estimated to be 35 megabase pairs.

**Table 1:** The four different GORD patient collections used in this study to identify collagen type III alpha I (COL3A1) as a susceptibility gene for GORD

Patient collection		Origin	Usage	Total no of individuals	No of patients with GORD	No of healthy individuals
1	Families	Women’s & Children’s Hospital Adelaide, Australia	Whole-genome linkage analysis	504	237	168
2	Trios	Women’s & Children’s Hospital Adelaide, Australia	Genetic association of candidate genes	1092	364	728
3	Case–control extended Kalixanda	Karolinska Institute, Sweden	Genetic association, replication of findings	741	256	485
4	Case–control EsoNerd	Sahlgrenska Hospital, Sweden	Immunohistochemistry	38	30	8
**Summary**				2375	887	1389

The family patient collection includes an additional 99 individuals with unknown gastro-oesophageal reflux disease (GORD) status. Affected children of the trios are listed as cases and their parents as controls. In the extended Kalixanda cohort, 98 individuals were diagnosed with both GORD and hiatus hernia (HH). The remaining 131 HH individuals were added from the original Kalixanda cohort,^28^ producing a separate HH case control material, consisting of 229 cases and the 485 controls.

Hu and colleagues previously identified linkage at chromosome 13q14 to GORD in a family material.^18^ None of our 36 families showed evidence for linkage to chromosome 13 (data not shown). We conclude that the 13q14 locus is not represented in our material, a finding in line with the results by Orenstein *et al*.^19^

### Genetic association analysis in the trio cohort

Oesophageal gene expression was used to identify candidate genes within the linkage region (Affymetrix chip U133A & B., EsoNerd study, Pierrou *et al*, manuscript in preparation). Three genes, CFLAR (CASP8 and FADD-like apoptosis regulator), COL3A1 (collagen type III alpha I) and ALS2CR2 (amyotrophic lateral sclerosis 2 (juvenile) chromosome region, candidate 2, also called STRADB), showed differential expression in oesophageal biopsies from patients with GORD compared with healthy controls. Genotyping of single nucleotide polymorphisms (SNPs) in the three genes in the 364 Trios, followed by genetic association analysis using transmission disequilibrium test (TDT), revealed significant association for SNPs only in COL3A1.

To establish that the association was confined to COL3A1, we analysed the linkage disequilibrium (LD) structure in the region by assaying multiple SNPs in COL3A1 as well as in the flanking genes DIRC1 (disrupted in renal carcinoma 1) and COL5A2 (collagen, type V, alpha 2). The LD block containing the disease-associated SNPs only harboured COL3A1. In males, we identified an associated haplotype in COL3A1 (p_corr_ of 0.00009; table 2) and an associated SNP (rs6434304) with a p_corr_ (p value adjusted for multiple testing) of 0.02 (table 3) . No single markers or haplotypes showed evidence for association in females in the Trio cohort.

**Table 2:** Haplotype analysis using single nucleotide polymorphisms (SNPs) covering collagen type III alpha I (COL3A1)

SNPs	Haplotype	Gastro-oesophageal reflux disease					
		Trios, all	Trios, males			Case–control, all	
		p Value	p Value	p_corr_	OR (95% CI)	p Value	OR (95% CI)
4:5	2:1	0.00096	0.00018	0.0026	3.00 (1.63 to 5.50)	0.0026	0.61 (0.43 to 0.85)
4:5	2:2	0.00019	0.000002	0.00009	0.40 (0.26 to 0.59)	NS	–
5–6	2:2	NS	NS	NS	No data	0.0041	1.42 (1.11 to 1.81)

Highly associated haplotypes were found in both the Trio cohort and in the adult case–control cohort. The association in Trios was found in males only.

CI, confidence interval; NS, not significant OR, odds ratio; p_corr_, p value adjusted for multiple testing.

**Table 3:** Genetic association of eight single nucleotide polymorphisms (SNPs) spread over collagen type III alpha I (COL3A1) in the Trio cohort and in the extended adult case–control cohort

Number	SNP	Gastro-oesophageal reflux disease					Hiatus hernia	
		Trios, all	Trios, males		Case–control, all		Case–control, males	
		p Value	p Value	p_corr_	p Value	p_corr_	p Value	p_corr_
1	rs12693520	0.18	0.0167	0.08	0.85	1.00	0.45	0.95
2	rs6434304	0.042	0.0037	0.018	0.95	1.00	0.25	0.76
3	rs2056156	0.21	0.0913	0.37	0.068	0.31	0.04	0.19
4	rs3106798	0.30	0.11	0.42	0.028	0.14	0.09	0.40
5	rs7579903	0.055	0.023	0.12	0.0037	0.022	0.44	0.94
6	rs1800255	0.092	0.068	0.31	0.44	0.95	0.09	0.37
7	rs1040187	0.95	0.76	1,0	0.14	0.54	0.38	0.90
8	rs3134646	0.91	0.39	0,91	0.025	0.13	0.003	0.019

Only the males from the Trio cohort show significant genetic association for SNP number 2 after multiple testing adjustments with a p_corr_ of 0.018. There is no gender bias in the adult case control cohort. Significant association for GORD in the case–control cohort was identified for SNP number 5 with a p_corr_ of 0.022. COL3A1 is also associated with hiatus hernia in the adult case–control cohort. The associated SNP, number 8, has a p_corr_ of 0.019. The identified association is male specific. The patient overlap is 98 individuals, eg, having both GORD and hiatus hernia.

### Genetic association analysis in the case–control cohort

Association analysis was repeated in the adult case–control cohort consisting of 256 GORD cases, 229 hiatus hernia cases, and 485 healthy controls. GORD and HH co-existed in 98 individuals. We used the same set of COL3A1 SNPs as previously, and were able to replicate the GORD association at a p_corr_ = 0.022 (table 3). No male specific association was observed.

The linkage to chromosome 2 originated from families selected for GORD and HH. To investigate if COL3A1 also was associated with HH, we stratified the case–control cohort for this phenotype and repeated the analysis. Significant association was identified in COL3A1. Interestingly, this was also male specific, with the most significant SNP (rs3134646) showing a p_corr_ = 0.019 (table 3). It was not possible to validate our finding in the paediatric trio cohort since HH typically develops after adolescence.

### Mutation analysis of COL3A1

Mutation detection was performed by polymerase chain reaction (PCR) amplification and DNA sequencing of all 51 exons of COL3A1 as well as 2 kb upstream of the transcription start site in 48 males and females from the trio cohort. These individuals represented both contributors and non-contributors with respect to the observed association. Intron–exon boundaries were included in the analysis. No amino acid changing mutation segregating with the disease was identified.

### Collagen type III protein expression analysis in oesophageal biopsies

The abundance of collagen type III was investigated in the epithelial lining of the distal oesophagus. Biopsies from GORD patients and healthy controls were obtained and stained with an antibody directed against human collagen type III. Patients had a significantly larger surface area staining than controls (p = 0.038 from 30 cases and eight controls. Thus, the data shows that collagen type III is upregulated in the epithelial lining of the oesophagus as a consequence of GORD. After gender stratification, the up regulation remained significant in males only (p = 0.033, 18 cases and 4four controls).

### Discussion

In the present study, based on four independent GORD patient collections (table 1), we show that COL3A1 is associated with GORD and implicated as being a risk factor for hiatus hernia (table 3). We identified linkage to the COL3A1 region in a set of families in which GORD and hiatus hernia is transmitted (fig 1). We show genetic association for COL3A1 to GORD in a paediatric Trio cohort and replicate the genetic association in an adult GORD case control cohort (tables 2 and 3). We also show genetic association between COL3A1 and hiatus hernia in the same adult case control cohort (table 3). Our genetic findings from these three independent patient materials are further strengthened by the protein studies from a fourth patient material, where the COL3A1 protein is up regulated in oesophageal tissue biopsies taken from GORD patients in comparison to healthy controls. We show that COL3A1 is associated to both GORD and HH but with different alleles. This becomes even clearer when comparing gender distribution where only males contribute to the HH association (table 3).

The gene COL3A1 encodes type III collagen, a fibrillar mono-trimeric extra cellular matrix protein that is present in extensible connective tissues such as skin, lung, and the vascular system, frequently together with type I collagen. Collagen type III has an important role in adjusting the strength and flexibility of tissues where it is expressed.^42^ Furthermore, COL3A1 is modulated in the wound response process. It is acutely upregulated in the early phases of wound healing and maintains high levels of expression for several weeks after injury. As healing progresses, collagen type III is replaced by collagen type I, leading to increased tissue strength.^43^

A number of COL3A1 mutations cause Ehlers–Danlos syndromes type III (EDS-III) and type IV (EDS-IV), both being autosomal dominant connective tissue disorders. Depending on the individual COL3A1 mutation, the severity of these diseases can vary from mild to life threatening. Symptoms include dermal manifestations, joint hyper-mobility and, in severe cases, spontaneous rupture of the bowel or large arteries.^44^ COL3A1 mutations similar to those underlying EDS are unlikely to be disease-causative in patients with GORD due to the milder phenotypic expression seen in GORD compared with EDS. One would rather expect mutations resulting in more subtle effects on gene function.

The genetic associations we see for haplotypes using the assayed SNPs (table 2) are much stronger than the genetic association seen for single SNPs (table 3). We conclude that none of the SNPs used in this study are causative but rather in linkage disequilibrium with the different disease alleles. We investigated the presence of causative mutations by sequencing the COL3A1 gene in 48 patients from the trio cohort. No disease-associated mutation, apart from the SNPs assayed, could be identified. Our interpretation is that the disease-causative mutations reside in regulatory sequences.

In our genetic analysis we identify several gender differences in the COL3A1 associations, both for GORD and HH. In the paediatric Trio cohort, only males showed association with GORD. In the adult case–control cohort, COL3A1 was associated with GORD without a gender bias, whereas only males showed association with HH. Finally, elevated collagen type III protein staining of the oesophageal biopsies from GORD patients was only found to be significant in males.

These differences can be attributed to differences between our materials such as: age, population origin, gender composition and disease definition. In fact, COL3A1 itself, is partly regulated by oestrogen.^45^ This is also shown by a the decline of collagen type III expression in post-menopausal women.^46^ Additionally, a gender difference in collagen deposition during wound healing has been reported in disease models.^47^ Furthermore, male gender and increasing age has been shown to be a risk factor for elevated wound healing times.^48^ Hence, the differences in association we observe in our analyses may be a consequence of the biological regulation of collagen type III.

From the data we have generated in the extended Kalixanda cohort it is clear that two different mechanisms are at work in COL3A1. One allele is associated with GORD while another allele confers male susceptibility to HH. The SNP that shows the strongest association with GORD (p_corr_ = 0.02) shows absolutely no evidence for association with HH (p_corr_ = 0.94). In this study we have an overlap of 98 patients where both GORD and HH are present. When testing if the male HH-associated SNP is also associated to GORD in males, the association is further weakened, underlining that there are two separate disease alleles for GORD and HH.

We hypothesise that an altered collagen type III expression in the oesophagus might lead to altered oesophageal tissue strength and flexibility, making the oesophageal lining prone to mucosal damage and wounds. Attenuated COL3A1 function may impair wound healing in the oesophagus. Mutations in COL3A1 have been shown to delay the wound healing response.^49^ An altered collagen type III expression could also contribute to an increased risk for developing HH. An altered collagen I/III ratio has been discussed in conjunction with HH.^50^ Hiatus hernia might be a risk factor for developing GORD through a mechanism involving increased distensibility of the oesophagogastric junction, resulting in GORD as a secondary event.^51^ Moreover, HH formation may lead to delayed gastric emptying, increasing the clearance time for acid refluxes in the oesophagus.^52^ We suggest that that the associated alleles of COL3A1 are conferring increased susceptibility to both GORD and HH, respectively.

We welcome a replication of our results from other groups to verify our findings of COL3A1 and, hopefully, identification of the causative mutations. We also raise the question whether there are differences in the wound healing response in controls versus individuals with HH due to an altered collagen I/III ratio.
